# Subtypes of Breast Cancer in Lao P.D.R.: A Study in a Limited-Resource Setting

**DOI:** 10.31557/APJCP.2019.20.2.589

**Published:** 2019

**Authors:** Thitsamay Luangxay, Siriphone Virachith, Kiyomi Hando, Soulideth Vilayvong, Phaengvilay Xaysomphet, Phetsamone Arounlangsy, Keokedthong Phongsavan, Makiko Naka Mieno, Naoko Honma, Masanobu Kitagawa, Motoji Sawabe

**Affiliations:** 1 *Department of Comprehensive Pathology, Graduate School of Medical and Dental Sciences,*; 9 *Department of Molecular Pathology, *; 3 *Department of Microbiology and Immunology, Graduate School of Health Care Sciences, Tokyo Medical and Dental University (TMDU), *; 8 *Department of Pathology, School of Medicine, Toho University,Tokyo,*; 4 *Department of Clinical Laboratory Sciences, Sanyo Women's College, Hiroshima,*; 7 *Center for Information, Jichi Medical University, Shimotsuke, Japan, *; 2 *Cancer Center, Mittaphab Hospital, *; 5 *Department of Pathology, University of Health Sciences,*; 6 *Department of Gynecology-Obstetrics, Setthathirath Hospital, Vientiane, Laos. *

**Keywords:** Breast cancer, low resources countries, immunohistochemistry, histological type, estrogen receptor

## Abstract

**Aim:**

The purpose of this study is to evaluate the prevalence of the immunohistochemical subtypes of breast cancer among Lao women by using immunohistochemistry (according to the St. Gallen 2017 guidelines) and to study their correlation to clinicopathological features in order to help guide better treatment plans for patients.

**Materials and methods:**

Formalin-fixed and paraffin embedded tissue blocks of 76 cases of primary invasive breast cancer were retrieved from the University of Health Sciences, Vientiane, Lao PDR, from 2013 to 2016. Patients’ information and previous histological reports were reviewed. Immunohistochemistry was done using antibodies against estrogen receptor (ER), progesterone receptor (PR), human epidermal growth factor receptor 2 (HER2/neu) and Ki-67 (MIB-1).

**Results:**

The mean age of the patients was 49 years, and the major histologic type was invasive ductal carcinoma, NOS (90.7%). The proportion of each subtype was hormone receptor-positive and HER2-negative, 44.7%; hormone receptor-positive and HER2-positive, 3.9%; hormone receptor-negative and HER2-positive, 13.2%; and triple-negative, 38.2%. ER was positive in 40.8% of the cases, while PR was positive in 47.4%. More than half of the cases were poorly differentiated cancer (65.8%), followed by moderately differentiated (34.2%). Tumors presented with pT2 (60.5%), followed by pT3 (25.0%) and pT4 (7.9%).

**Conclusion:**

Breast cancer among Lao women is characterized by a large percentage of the triple-negative subtype that is less susceptible to hormonal treatments. The empirical treatment with tamoxifen should be reconsidered since it would be less effective to these patients. More importantly, basic pathology services should be the first requirement in Lao PDR in order to provide adequate care.

## Introduction

Breast cancer is the second most common cancer among the female population around the world (http://www.who.int/cancer/detection/breastcancer/en/index1.html, accessed June 18, 2018). Despite the recent advances in the therapeutic area, many low-resource countries still have very limited access to treatments. 

Lao PDR is one of the fastest growing economies in East Asia and the Pacific according to the World Food Program (http://www1.wfp.org/countries/lao-peoples-democratic-republic, accessed June 18, 2018), but the development in women’s health ranks the worst. With a population of 6 million, the healthcare system still struggles to provide adequate care for the whole population. The critical lack of healthcare professionals, especially in pathological and oncological sections, might be problematic for the care of patients. Regardless of the current circumstances, histopathological diagnosis should be the minimum requirement to diagnose breast cancer. This should include immunohistochemical testing for estrogen receptor (ER), progesterone receptor (PR) and human epidermal growth factor receptor 2 (HER2) (El Saghir et al., 2011).

Like many other low-resource countries, the lack of infrastructure of healthcare facilities is the reason why these three main treatment markers are not routinely tested. Quality control to validate these tests is also unavailable, and patients or their families have to pay for additional examination costs (Harford et al., 2008). Without hormonal receptor status and HER2 status, treatment decisions are based on clinical findings. Therefore, it is almost impossible to select the patients who will benefit from hormonal therapy or Herceptin.

Currently, none of the related research that has been published is about Laos. The lack of these data makes it impossible to predict patients’ responsiveness to treatments. Most patients present with locally advanced disease with axillary lymph node metastasis.

Our available treatments would cause minimal impact in this particular group of patients. 

In addition to the poor healthcare structure in most low-resource countries, the perception of the patient that the treatment may not be working for this type of disease might contribute to a delay in receiving treatment until no further options are available.

Despite advances in molecular techniques, the detection of hormonal receptors and HER2 is still done by immunohistochemistry in many laboratories. These three basic markers remain the most important predictive factors without substantial change since they were first used (Rakha and Green, 2017). Therefore, in this study, the tumor subtypes will be stratified according to St. Gallen International Expert consensus 2017 (Curigliano et al., 2017) regarding hormonal status and HER2. Even though the American Society of Clinical Oncology/College of American Pathologist (ASCO/CAP) has not endorsed the Ki-67 proliferation index to be a guideline for adjuvant chemotherapy because of the lack of expert consensus (Andre et al., 2015), we nonetheless also examined this marker. Therefore, the aim of this study was to evaluate the prevalence of the immunohistochemical subtypes of breast cancer among Lao women by using immunohistochemistry (ER, PR, HER2 and Ki-67) and to study their correlation to morphological features.

## Materials and Methods

*Patient database*

This is a retrospective study done at the University of Health Science, Department of Pathology, located in Vientiane, the capital city of Laos. This department has been serving the population of Vientiane, where most of the breast cancer diagnoses were made, with only 2 pathologists and 2 medical technologists currently working there. The formalin-fixed and paraffin embedded tissue blocks were retrieved from 76 cases with primary invasive breast cancer from 2013 to 2016. Patients’ information and previous histological reports were reviewed. Hematoxylin-eosin slides were prepared from these blocks, and the diagnoses of invasive breast carcinoma were made with the adequate supportive information. The slides were reevaluated for histological type and grade by using the Nottingham Histologic Score (Bloom and Richardson, 1957; Rakha et al., 2010) along with the correlation to clinical information.

*Immunohistochemistry*

ER/PR: Immunohistochemistry was done using anti-human estrogen receptor (ER α, clone EP1, code IR084, Ready to use, Agilent Technologies Japan, Ltd., Tokyo, Japan) and anti-human progesterone receptor (PR, clone PgR636, code IR068, Ready to use, Agilent Technologies Japan, Ltd., Tokyo, Japan). Peroxidase (3% H_2_O_2_/DW) was used for nonspecific blocking. For antigen retrieval, the sections were heated in a warm bath at 95°C for 20 min in Target Retrieval Solution at pH 9 (EnVision ™ FLEX, High pH (50x), code GV804, Agilent Technologies Japan, Ltd., Tokyo, Japan), followed by cooling to room temperature. Primary antibodies were applied and incubated in a moist chamber for 30 min at room temperature for each antibody. The second antibody was applied using labeled polymer (EnVision+ System- HRP, code K4002, Agilent Technologies Japan, Ltd., Tokyo, Japan) and incubated in a moist chamber for 30 min. To visualize the positivity, DAB chromogen was used with Mayer Hematoxylin counter stain. ER and PR positive controls were added for each immunostaining.

Ki-67: By using the similar protocol for antigen retrieval for ER/PR described above, the sections were heated in a warm bath at 95°C for 40 min in Target Retrieval Solution at pH 9. Ki-67 antigen (Clone MIB-1, code M7240, Agilent Technologies Japan, Ltd., Tokyo, Japan) was diluted to 1:100 then applied as the primary antibody and incubated in a moist chamber for 30 min at room temperature. We used the same second antibody as for ER/PR for detecting bound primary antibodies, and we used DAB to examine the positivity of the staining.

HER2: For detecting human epidermal growth factor 2, Histofine® HER2 Kit MONO (clone SV2-61γ, Code 427041, Nichirei Biosciences, Tokyo, Japan) was used according to the manufacture instructions. All sections were digested with protease solution at room temperature for 5 min. Then, the nonspecific activity was blocked with 3% hydrogen peroxide for 5 min. The anti-HER2 antibody was applied at 5 µg/mL at room temperature for 30 min, followed by application of goat anti-mouse Fab’ antibody for 30 min. We applied DAB (3,3’-diaminobenzidine) and incubated for 10 min in order to see the positivity. Positive and negative controls were applied in each staining session. 

*ER/PR, HER2 scoring assessment and Ki-67 cut-off value*

The slides were considered positive if there was at least 1% of the nuclei of the invasive cancer cells stained and negative if the nuclear staining was completely absent (Hammond et al., 2010). HER2 scores were assessed by using the standard ASCO/CAP guideline reporting system on a score scale of 0, 1+, 2+ and 3+. With a score of 3+, the section was considered positive and showed strong, complete membrane staining of more than 10% of the invasive tumor cells. HER2 testing by fluorescence in situ hybridization (FISH) was not performed in this study. The Ki-67 cut-off value was divided into three groups: low (≤ 15%), intermediate (16-30%) and high (>30%) based on a previous study (Inwald et al., 2013).

*Ethical clearance*

This research was approved by the University of Health Sciences Ethics Committee (Ref no. 6685/2016) and the Tokyo Medical and Dental University Ethics Committee (Ref no. M2017-049).

*Statistical analysis*

ANOVA was used to compare the age of the patients among four subtypes of breast cancer. Fisher’s exact tests were employed for comparing the clinicopathological features among these groups. All statistical analyses were done by SAS (Version 9.4, SAS Institute Inc., Cary, NC, USA). P values less than 0.05 were considered statistically significant.

## Results

*1. Patient profiles*

The mean age was 48 years (range 24 – 76 years), with 2 cases of unknown age, but both of them were documented as adults. More than half of patients (55.4%) were less than 50 years of age. A majority (70 cases) were invasive ductal carcinoma not otherwise specified (NOS) (92.1%). The remaining histological types included two cases of invasive lobular carcinomas (2.6%), three of medullary carcinoma and one of invasive micropapillary carcinoma (5.3%). Overall, 65.8% were grade III, 34.2% were grade II, and no grade I tumors were identified (Table 1). The majority showed tumor size between 2 to 5 cm (60.5%). Most patients underwent total mastectomy with axillary lymph node dissection (modified radical mastectomy) (88.2%). Half of them presented with axillary lymph node metastasis at the time of diagnosis (52.6%).

*2. Immunohistochemical profile*

**Figure 1:**
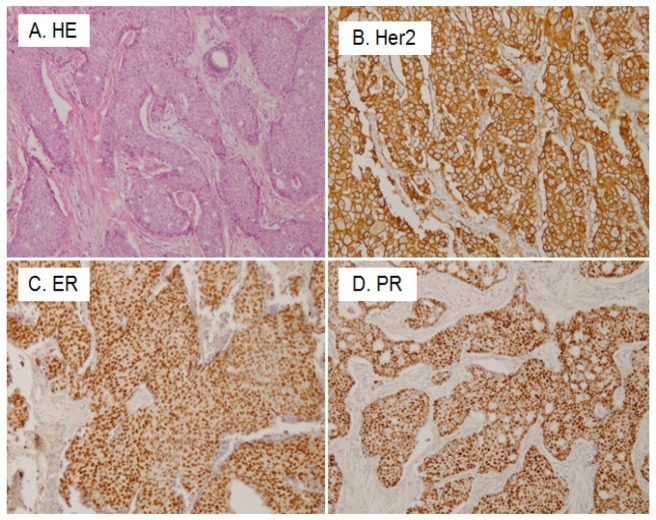
Histological and Immunohistochemical Results of Invasive Ductal Carcinoma of the Breast. A, Histological picture of the cancer; B, Human epidermal growth factor receptor 2 (Her 2) immunostaining. The cellular membrane of cancer cells are strongly positive (3+); C, Estrogen receptor (ER) immunostaining. The nuclei of cancer cells are positive; D, Progesterone receptor (PR) immunostaining. The nuclei of cancer cells are positive

The representative pictures of immunohistochemistry are shown in Figure 1. Hormonal receptor and HER2 positivity were as follows: ER in 40.8%, PR in 47.4% and HER2 in 17.1%. The subtype classification showed hormone receptor-positive and HER2-negative, 44.7%; hormone receptor-positive and HER2-positive, 3.9%; hormone receptor-negative and HER2-positive, 13.2%; triple-negative, 38.2%.

*3. Clinicopathological features in each subtype of breast cancer*

Grade III tumors were frequently seen in triple-negative and HER2-positive subtypes (p < 0.001) but did not show any relation with higher tumor size or nodal metastasis. The mean Ki-67 index was 25.7% (±SD, 25.7 ±29.1), with a median score of 11.5% (ranging from 1 - 95%).

## Discussion

We collected 76 cases of primary breast cancer at UHS in Vientiane, Laos. The women were relatively young, with a mean age of 48 years. Although the menopausal state was not known, more than half (55.4%) were less than 50 years of age. Approximately 60% of the patients presented 2-to-5 cm-sized large tumors, and more than half (52.6%) showed nodal metastasis. ER, PR and HER2 was positive in 40.8%, 47.4% and 17.1% of the cases, respectively, and triple-negative cases comprised 38.2%.

**Table 1:** Comparison of the Clinicopathological Features Among the Four Subtypes of Breast Cancer

	Total	HR+/HER2−	HR+/HER2+	HR−/HER2+	Triple-negative	P-Value
No. of Cases (%)	76	34 (44.7)	3 (3.9)	10 (13.2)	29 (38.2)	
Age	49.1 ± 10.9	47.3 ± 10.1	50.0 ± 3.0	56.2 ± 8.5	48.9 ± 12.5	0.19
Tumor Grade						0.001
Grade II	26 (34.2)	19 (55.9)	0 (0)	0 (0)	7 (24.1)	
Grade III	50 (65.8)	15 (44.1)	3 (100)	10 (100)	22 (75.9)	
Tumor size						0.62
≤ 2 cm (T1)	5 (6.6)	2 (5.9)	0 (0)	0 (0)	3 (10.3)	
< 2 cm – 5 cm ≤ (T2)	46 (60.5)	22 (64.7)	1 (33.3)	7 (70.0)	16 (55.2)	
> 5 cm (T3)	19 (25.0)	6 (17.7)	2 (66.7)	2 (20.0)	9 (31.0)	
Involving skin and chest (T4)	6 (7.9)	4 (11.7)	0 (0)	1 (10.0)	1 (3.5)	
Nodal status						0.69
Positive	40 (52.6)	18 (53.0)	2 (66.7)	6 (60.0)	14 (48.3)	
Negative	24 (31.6)	8 (23.5)	1 (33.3)	3 (30.0)	12 (41.4)	
Unknown	12 (15.8)	8 (23.5)	0 (0)	1 (10.0)	3 (10.3)	
Ki-67 index						0.11
Low	41(53.9)	21 (61.8)	1 (33.3)	3 (30.0)	16 (55.2)	
Intermediate	9 (11.8)	6 (17.6)	0 (0)	0 (0)	3 (10.3)	
High	26 (34.2)	7 (20.6)	2 (66.7)	7 (70.0)	10 (34.5)	

* HR, Hormonal receptor; HER2, Human epidermal growth factor receptor 2

*Hormone receptor-positive breast cancer*

This study shows that 40.8% of these tumors were positive for estrogen receptor. This prevalence is lower than that in other neighboring South east Asia countries, such as Thailand (71.6%) (Chuthapisith et al., 2012), and Myanmar (57.1%) (San et al., 2017), and slightly lower than Cambodia (48.3%) (Ley et al., 2016). This would suggest that Lao women may respond less to the empirical tamoxifen or aromatase inhibitors, since fewer than half would benefit from endocrine therapy. Regarding the breast cancer subtype, the prevalence of hormone receptor-positive cases was 44.7%, which is much lower than the results found in other developed countries in Asia, Europe and the US: 71% in Japan (Shibuta et al., 2011), 62% in Taiwan (Lin et al., 2009), 72% in Europe (Blows et al., 2010), and 72.7% in the US (Howlader et al., 2014).

Our patients presented at young ages, with a mean age of 48 years old, and they tended to have higher histologic grade, and this trend can be observed in a recent study (Copson et al., 2018). The mean age is high in other developed countries in Asia, such as Japan (56 years) and Taiwan (50 years). The younger patients’ presentation may not reflect the overall presentation of breast cancer in Lao women. Laos seems to be a society where older women seldom seek further treatment if they determine that they have an advanced breast cancer because they believe that its treatment unavailable, and it would also put burdens on the rest of the family. Thus, not seeking care for their cancer is one of the final options for them. That might explain why our patients tended to be younger because young patients believe that they would have a better chance to survive than older women. As stated in the study of the Breast Health Global Initiative (BHGI) (El Saghir et al., 2011), cultural beliefs, poverty, the lack of breast cancer awareness and the level of education play major roles in patients choosing not to seek treatment. 

*HER2-positive breast cancer*

The hormone receptor-negative and HER2-positive subtype in our study shows the same frequency as the study in Thailand (13.2% vs. 13.3%), which reported that HER2 overexpression tended to be more frequent in Thai women than in US women (Chuthapisith et al., 2012). This could raise the possibility of the aggressiveness of breast carcinoma in the neighboring country of Laos, which might share a similar ethnic background; however, the prevalence of the HER2-positive subtype differs around Asia, ranging from 6% to 65%, with the median being 19% by immunohistochemistry for score 3+ cases and 25% by FISH (Tan et al., 2010). Unfortunately, we could not perform FISH in our HER2 score 2+ cases. Many studies have confirmed the aggressiveness and poor prognosis of HER2-positive breast cancer with strong evidence (Vaz-Luis et al., 2012). It is to be noted that the access to anti-HER2 targeted therapy is limited or nonexistent in areas with low levels of healthcare resources.

*Triple-negative breast cancer*

Triple-negative breast cancer showed a remarkably high percentage (38.2%) in our study. The excess of the triple-negative subtype could be a hidden genetic factor common among Lao women, but no genetic study has ever been carried out before. Furthermore, high-grade tumors showed a significantly higher percentage in our study (65.8%). This could mean that breast cancer among Lao women tends to be aggressive in nature and occurs in young patients. Nonetheless, we cannot truly confirm whether these women truly present with aggressive tumors. This significantly high rate of triple-negative breast cancer requires our attention because the possibility of a false high rate of hormonal receptor negativity should be carefully considered, caused by the poor tissue preparation, inadequate duration and quality of the fixation, and the interpretation of the finding. This issue was already mentioned in several retrospective studies in Africa, which showed high rates of hormonal receptor-negative carcinoma (Adebamowo et al., 2008; Bird et al., 2008). Another study found that the variety of molecular subtypes of breast carcinoma was associated with different survival rates and that the survival was the lowest among the HER-2 overexpressed and triple-negative groups, even after consecutive treatment (Kongsiang et al., 2015). In another study in a low-resource setting, triple-negative breast carcinoma occupied a greater proportion in Uganda women (36%) (Roy and Othieno, 2011) compared to those in European American women and African American women (21% and 24%) (Morris et al., 2007; Stark et al., 2008). This report suggested that Ugandan women had a greater number of triple-negative breast cancer cases, which was accompanied with poor prognosis. This triple-negative phenotype of breast cancer, common among African American women, is strongly associated with BRCA1 mutations and is more likely to resist hormone therapy with an aggressive behavior (Pal et al., 2015). To highlight this, we need to perform more in-depth studies among our triple-negative breast cancer population. 

*Single hormone receptor-positive*

There was a small number of ER- PR+ (7.8%) cases in our study; this finding was similar to a study in Cambodia (9.2%) (Ley et al., 2016). Interestingly, Ng (2012) stated that this phenotype of breast cancer was common among premenopausal young women (Ng et al., 2012) and tended to have more aggressive clinical behavior than hormonal receptor-double positive tumors (ER+/PR+). In our study, this phenomenon was found in women aged less than 50 years. In 2015, an interesting finding from the study of Chan (2015) suggested that this subtype might arise from the failure of anti-ER antibody to bind ER, which was caused by structural changes due to genetic mutations (Chan et al., 2015), although it might be simply ascribed not to the genetic change of cancer cells but rather to technical error. 

*Breast cancer awareness*

Socioeconomic factors and cultural beliefs may contribute to the late seeking of cancer care. As some women believe that there is no cure for breast cancer, they pay no attention to the growth of breast cancer until the final stage. The lack of awareness of breast cancer in the local community causes a barrier to early detection and treatment (El Saghir et al., 2011). Although most Lao women presented in the locally advanced stage (85.5%) in our study, early detection and care are still recommended to reduce the cost and duration of therapy. Additionally, even if the hormonal receptor status is known, the cost of hormonal therapy is relatively high for most of the patients; hence, they ‘run away’ from the current treatment course. The discontinuity of the course has effects on low therapeutic efficacy and final loss of the patients during follow-up. The lack of early breast cancer screening programs and healthcare education among Lao women might be the main contributor to the late presentation of breast cancer patients. Half of the patients presented with lymph node involvement at the time of the surgery (52.6%) in this study. This rarely happens in developed countries where the screening programs are high-functioning. The Ministry of Health of Thailand recommends that women aged 25 years and older have knowledge of breast self-examination. Although this cannot reduce the mortality rate from breast cancer, it would increase the self-awareness to get regular breast checkups, such as mammography (Tassanasunthornwong et al., 2015). Laos should also promote this model, since breast cancer screening cannot cover the whole population, and women might have to pay to get tested. 

## Limitations

In this retrospective study, there was no follow-up information on the patients, such as postoperative therapy and prognosis. The quality control of the specimens may not have met the standard criteria. The poor preparation of tissue fixation and tissue processing can affect immunohistochemistry reliability. Thus, the interpretation of these data should be carefully considered. We excluded unsuitable paraffin blocks, which reduced the number of cases available for analysis and made some statistical tests inapplicable.

Furthermore, the findings of this study may not be generalizable for the entire female population of Lao PDR since we analyzed surgical samples that were received only from the Department of Pathology, UHS, in Vientiane capital, where most of the patients were treated in Setthathirath Hospital, which is the only hospital that can offer treatment for breast cancer, such as surgery, hormonal therapy and chemotherapy.

In conclusion, breast cancer among Lao women is characterized by a large percentage of the triple-negative subtype that is less susceptible to hormonal treatments. The empirical treatment with tamoxifen should be reconsidered since it might be less effective for these patients. HER2-positive and triple-negative breast cancer need to be further investigated. Basic pathology services should be the first requirement in Lao PDR in order to provide adequate care for cancer patients. Our findings could provide fundamentally useful data for national policies in order to control breast cancer in the future.

## Statement of conflict of interest

The authors declare no potential conflicts of interest with respect to the research, authorship, and/or publication of this article.
